# Of the Koumiss of the Calmucks, and of the Ardent Spirit Which They Distil from Milk

**Published:** 1811-03

**Authors:** Edward Daniel Clarke


### *Of the Koumiss of the Calmucks, and of the ardent Spirit which they distil from Milk. By* Edward Daniel Clarke, *LL.D.*

[Clarke's Travels in Europe, Asia and Africa.]

EVERY body has heard of the koumiss and the brandy which the Calmucks are said to distil from the milk of mares. The manner of preparing these liquors has been differently related, and, perhaps, is not always the same. They assured us that the brandy was merely distilled from butter-milk. The milk which they collect over night is churned in the morning into butter; and the butter-milk is distilled over a fire made with the dung of their cattle, particularly the dromedary, which makes a steady and clear fire, like peat. But other accounts have been given both of the koumiss and the brandy. It has been usual to confound them, and to consider the koumiss as their appellation for the brandy so obtained. By every information I could gain, not only here, but in many other camps which we afterwards visited, they are different modifications of the same thing, although different liquors: the koumiss being a kind of sour milk, like that so much used by the Laplanders, called *pina*, and which has undergone, in a certain degree, vinous fermentation; and the brandy, an ardent spirit obtained from koumiss by distillation.

In making the koumiss they sometimes employ the milk of cows, but never if mares milk can be had; as the koumiss from the latter yields three times as much brandy as that made from cow's milk.

The manner of preparing the koumiss is by combining one-sixth part of warm water with any given quantity of warm mare's milk. To this they further add, as a leaven, a little old koumiss, and agitate the mass till fermentation ensues.

ensues. To produce the vinous fermentation, artificial heat and more agitation are sometimes necessary. This affords what is called koumiss.

They gave us this last beverage in a wooden bowl, calling it *vina*. In their own language it bears the very remarkable appellation of rack and racky, doubtless nearly allied to the names of our East-India spirit, rack and arrack. We brought away a quart bottle of it, and considered it very weak bad brandy, not unlike the common spirit distilled by the Swedes and other northern nations.

Some of their women were busy making it in an adjoining tent.

The simplicity of the operation and of their machinery was very characteristic of the antiquity of this chemical process. Their still was constructed of mud, or very coarse clay; and for the neck of the retort they employed a cane. The receiver was entirely covered by a coating of wet clay. The brandy had already passed over. The woman who had the management of the distillery, wishing us to taste of the spirit, thrust a stick with a small tuft of camel's hair at its end, through the external covering of clay: and thus collecting a small quantity of brandy, she drew out the stick, dropped a portion upon the retort, and, waving the instrument above her head, scattered the remaining liquor in the air. I asked the meaning of this ceremony; and was answered, that it is a religious custom, to give always the first drop of the brandy which they draw from the receiver to their god. The stick was then plunged into the receiver a second time; when more brandy adhering to the camel's hair, she squeezed it into the palm of her dirty greasy hand, and, having tasted the liquor, presented it to our lips. . . . .

We traversed continued *steppes* [immense flats] from Kamenskaia. Camps of Calmucks were often stationed near the road. We paid visits to several of them. In one of them, containing not more than four tents, we found only women, who were busy in distilling brandy from milk. The women confirmed what we had been before told concerning the materials used for distilling, and said that, having made butter, they were distilling the butter-milk for brandy. We could not credit that brandy might be so obtained; but to prove it, they tapped the still as upon a former occasion, offering us a tuft of camel's hair soaked in brandy, that we might taste, and be convinced.

